# Componential usage patterns in dengue 4 viruses reveal their better evolutionary adaptation to humans

**DOI:** 10.3389/fmicb.2022.935678

**Published:** 2022-09-20

**Authors:** Gun Li, Liang Shi, Liang Zhang, Bingyi Xu

**Affiliations:** ^1^Laboratory for Biodiversity Science, Department of Biomedical Engineering, School of Electronic Information Engineering, Xi'an Technological University, Xi'an, China; ^2^Key Laboratory of Analytical Chemistry for Life Science of Shaanxi Province, School of Chemistry and Chemical Engineering, Shaanxi Normal University, Xi'an, China

**Keywords:** codon usage pattern, dengue 4 virus, gene evolution, adaptive evolution, evolutionary trend

## Abstract

There have been at least four types of dengue outbreaks in the past few years. The evolutionary characteristics of dengue viruses have aroused great concerns. The evolutionary characteristics of dengue 4 viruses are studied in the present study based on their base usage patterns and codon usage patterns. The effective number of codons and relative synonymous codon usage (RSCU) values of four types of dengue viruses were counted or calculated. The Kullback–Leibler (K–L) divergences of relative synonymous codon usage from dengue viruses to humans and the Kullback–Leibler divergences of amino acid usage patterns from dengue viruses to humans were calculated to explore the adaptation levels of dengue viruses. The results suggested that: (1) codon adaptation in dengue 4 viruses occurred through an evolutionary process from 1956 to 2021, (2) overall relative synonymous codon usage values of dengue 4 viruses showed more similarities to humans than those of other subtypes of dengue viruses, and (3) the smaller Kullback–Leibler divergence of amino acid usage and relative synonymous codon usage from dengue viruses to humans indicated that the dengue 4 viruses adapted to human hosts better. All results indicated that both mutation pressure and natural selection pressure contributed to the codon usage pattern of dengue 4 viruses more obvious than to other subtypes of dengue viruses and that the dengue 4 viruses adapted to human hosts better than other types of dengue viruses during their evolutionary process.

## Introduction

Dengue fever is a mosquito-borne viral disease that has rapidly spread in many countries. The World Health Organization summarized the dengue fever and the severe dengue fever pandemics in recent years, pointing out that even today, the dengue viruses may affect most Asian and Latin American countries (http://www.who.int/mediacentre/factsheets/fs117/en/). More and more people are at risk for there is no specific treatment for dengue viruses (Wong et al., 2020). The global incidence of dengue viruses has grown dramatically, for instance, according to the US Centers for Disease Control and Prevention (CDC), as of 5 January 2022, there were 117 dengue fever cases reported to the CDC by state, territorial, and local health departments during 2021, and according to the WHO, a total of 48,906 dengue fever cases including 183 deaths, have been reported in Pakistan from 1 January to 25 November in 2021. There are at least four distinct serotypes, i.e., dengue 1 (Colavita et al., 2020), dengue 2 (Singh et al., 2017), dengue 3 (Wang et al., 2017), and dengue 4 (Veronica et al., 2020) of viruses that could cause dengue fever. Study on these four distinct serotypes and other emerging serotypes of dengue viruses (Mustafa et al., 2015) that appeared recently, for instance, dengue viruses emerged in China (Yue et al., 2019), Russia (Sergeeva et al., 2015), India (Annette et al., 2016), Malaysia (Shueb et al., 2016), South America (Cruz et al., 2016), Indonesia (Agus et al., 2006; Sasmono et al., 2015), and in mosquitoes (Santos et al., 2017), etc. (Cortes et al., 2018; Xu et al., 2019), have already attracted great attentions (Chai et al., 2022; Saud et al., 2022).

Codon usage bias (CUB) is due to the degenerate usage of synonymous codons for coding one certain amino acid, which exists in all organisms. Evolutionary changes and adaptive fitness to hosts of viruses could be reflected *via* their codon usage patterns (Siddiq et al., 2022). The CUB is governed by many factors, such as genetic mutation (Li et al., 2021), genetic drift and selection pressure (Gajbhiye et al., 2017), tRNA abundance, and sequence length. Exploring the CUB extent and its contributing factors in viruses may provide clues to reveal the adaptive degree to hosts. Many previous studies focused on the relationship between gene expression and CUB, and on exploring the molecular evolution mechanisms by comparing the relative synonymous codon usage (RSCU) values among different species (Khandia et al., 2019). From the biochemical respective, viruses depend on their hosts to survive as most of them do not have their own tRNAs. Therefore, viruses must optimize their biochemical compositions to adapt to the hosts during their evolutionary process, and the similarity of codon usage pattern between viruses and hosts were compared to determine the adaptation of viruses (Tian et al., 2018). However, recent studies have revealed that patterns of codon usage within many viral genomes are far more complex than previously imagined, and the factors shaping their evolution are still not entirely understood (Chakraborty et al., 2019). Therefore, some methods were designed to explore the serotypes of the dengue virus (Christenbury et al., 2010; Buchillet, 2012), for instance, an effective method for recognizing the multiple antigen peptides for mimotope of the dengue 3 viruses was designed (Amin et al., 2016), and a traditional phylogenetic analysis was also used for studying the genotype replacement among dengue viruses (Suzuki et al., 2019). All previous studies have not mentioned the adaptive fitness of dengue viruses. To investigate the hypothesis that human hosts could lead to CUB variations in dengue viruses, and to better elucidate the adaptive evolutionary characteristics of the dengue viruses, we calculated the differences in the base usage pattern, the guanine-cytosine content (GC) content, the effective number of codons (ENCs), the RSCU values, the amino acid usage pattern between humans, and the four types of dengue viruses.

## Materials and methods

All dengue 4 virus genomes deposited in the National Center for Biotechnology Information (NCBI; http://www.ncbi.nlm.nih.gov) database were considered in the present study. Among them, dengue 4 virus genomes from nonhuman hosts (i.e., OK605599.1 from African green monkey, JF262779.1, and JF262780.1 from sentinel monkey, and KY451945.1 and MN192436.1 from *Aedes aegypti*) were excluded (Thikhumporn et al., 2017). At last, a total of 205 selected sequences of *Homo sapiens* hosts were included in the present study (Supplementary File 1). Componential properties including G, C, A, and U of dengue 4 virus genomes were calculated and compared with those of humans to further evaluate their adaptive evolutionary characteristics. As a comparison, other sub-types of dengue viruses of human hosts were also performed similarly.

### ENC analysis

Effective number of codons analysis, as an important method for measuring the codon usage pattern, and can be used to quantify the codon usage bias within genes. It can be calculated by the following equation (Wright, 1990):


(1) $$ENC^{calculatied}=2+\frac{9}{{\bar{f}}_{2}}+\frac{1}{{\bar{f}}_{3}}+\frac{5}{{\bar{f}}_{4}}+\frac{3}{{\bar{f}}_{6}}$$

where ${\bar{f}}_{k}$(*k* = 2, 3, 4, 6) in the equation is the mean of *f*_*k*_ for the *k*-fold degenerate amino acids, and the value *k* in the ${\bar{f}}_{k}$ also denotes the average homozygosity for the amino acid class of *k* codon degeneracy. The coefficients number 9, 1, 5, and 3 in the equation denote the number of amino acids belonging to different degeneracy classes. Here, *f*_*k*_ could be calculated *via* the formula:


(2) $$\begin{array}{r} f_{k}=\frac{n\text{s}-1}{n-1} \end{array}$$

where *n* is the total number of occurrences of the codons for certain amino acid and the *s* could be calculated *via* the following equation.


(3) $$\text{s}={\sum_{i=1}^{k}\left(\frac{n_{i}}{n}\right)}^{2}$$

where *n*_*i*_ in the equation (3) is the number of the *i*-th codon for that certain amino acid. If the codon choice was only constrained by a mutation bias, the ENC value of the gene would lie on or just below the curve of the expected (Li et al., 2022). The expected ENC value should obey the rule ENC^*expected*^ = 2 + *s* + 29/[*s*^2^+ (1 – *s*)^2^]. The ENC analysis was usually used to quantify the absolute codon usage bias by evaluating the degree of codon usage bias in coding sequences. Therefore, to elucidate the effect of human hosts to the genetic characteristics of the dengue 4 viruses, the values of related parameters for humans were further calculated based on the coding sequences within human chromosomes (NC000001–NC000024) (Supplementary File 2).

### Neutral evolution analysis

The neutrality plot is also called the neutral evolution analysis, which could be performed to determine and compare the influence extent of mutation pressure. In this study, the neutral evolution analysis on the codon usage patterns of dengue 4 viruses by considering the value GC_12_/(GC_12_ + AU_12_) against the GC_3_/(GC_3_ + AU_3_) was conducted and analyzed by plotting the GC_12_ ratio for all sequences of dengue 4 viruses. In the plotting, the ordinate is shortened to GC_12_, and the abscissa is shortened to GC_3_.

### RSCU analysis

The RSCU values of all genes in dengue 4 viruses were calculated to explore the synonymous codon usage *via* a previously described method (Cristina et al., 2015).


(4) $$RSCU=\frac{g_{ij}}{\sum_{j}^{n_{i}}g_{ij}}n_{i}$$

where *g*_*ij*_ is the observed number of the *i*-th codon for the *j*-th amino acid, which has *n*_*i*_ kinds of different synonymous codons. The synonymous codons with RSCU values >1.0 are usually regarded as abundant codons, whereas those with RSCU values <1.0 are defined as less-abundant codons. Based on the codon usage frequencies of the genomes of dengue 4 viruses, the RSCU values for dengue 4 viruses were further used to calculate the effect of the hosts on that of dengue 4 viruses.

### Codon bias index (CBI) analysis

The codon bias index (CBI) values could reflect the presence of components with high codon usage in a particular gene. The CBI value can clearly describe the foreign gene expression in the host. It has been widely used to express the codon bias. The CBI value can be calculated by the following formula (Nathchoudhury et al., 2017):


(5) $$\begin{array}{r} CBI=\frac{N_{opt}-N_{ran}}{N_{tot}-N_{ran}} \end{array}$$

where the *N*_*opt*_ represents the total number of occurrences of the superior codon in the gene, in this work, the superior codons were the codons whose RSCU value was more than 1.6. *N*_*ran*_ represents the sum of the number of occurrences of the superior codon when all the synonymous codons are random in a certain protein; *N*_*tot*_ represents the occurrence number of the amino acid corresponding to the superior codon in a gene. To explore the relationship between the CBI and the ENC for dengue 4 viruses, the ENC values were equalized to the range of the CBI *via* the following equation:


(6) $$\begin{array}{r} \text{E.ENC}=\frac{ENC-ENC_{\min}}{ENC_{\max}-ENC_{\min}}\left(CBI_{\max}-CBI_{\min}\right)+CBI_{\min} \end{array}$$

Differences in RSCU values between genomes can be used to describe their evolutionary distance. The Kullback–Leibler (K–L) divergence between dengue 4 viruses and humans were also analyzed from both the RSCU and the amino acid ratio level by calculating their relative entropy. The K–L divergence, which is also named as the relative entropy, could be calculated by the formula (Wen et al., 2019):


(7) $$KL(P||Q)=\sum P(x)\text{log}2\left(\frac{P(x)}{Q(x)}\right)$$

where *KL(P||Q)* is also defined as information divergence, representing the degree of similarity between virus and host, while the *P* and *Q* in the formula represent the RSCU values of viruses and the human hosts, respectively. When the K–L divergence of RSCU values was calculated, 3 terminal codons, codons for Met and Trp were excluded; therefore, there were 59 synonymous codons concerned. *KL(P||Q)* represents the potential effect of hosts on viruses from overall codon usage and amino acid ratio, and the value ranges from 0 to 1 (Agahi, 2021). Codon bias analysis is very important for exploring the evolution of viruses from the molecular level. All calculations in the present study were performed on the Matlab R2010b software by an in-house script.

## Results

### Componential usage of dengue 4 viruses

**Figure 1:**
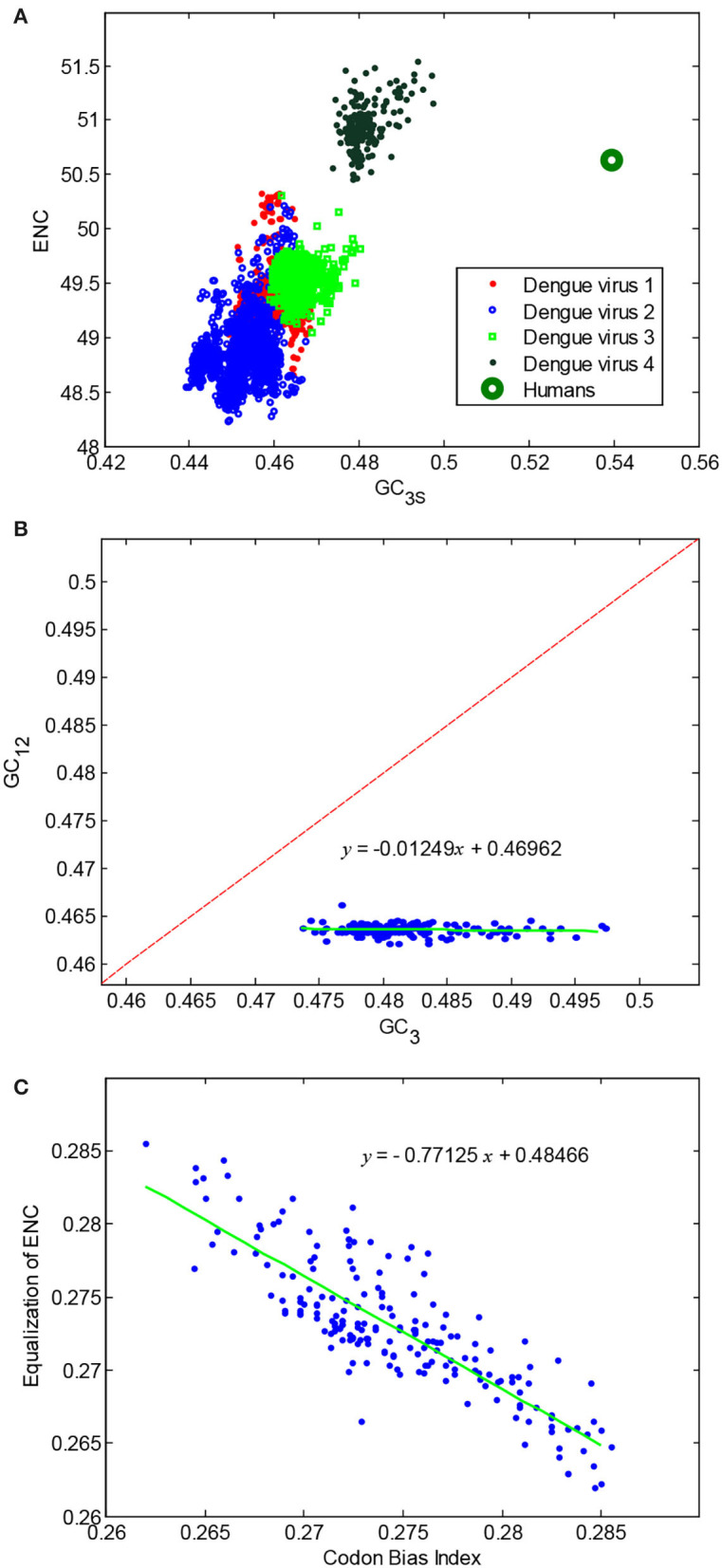
Componential usage pattern in dengue 4 viruses. **(A)** The effective number of codons (ENCs) plot of four types of dengue viruses; **(B)** the neutral plot of dengue 4 viruses; and **(C)** equalization of ENC vs. codon bias index (CBI) of dengue 4 viruses.

Base composition and codon usage patterns, such as the GC_3_, GC_12_, overall GC content, ENC, and CBI, which would affect the codon usage patterns of a genome, of dengue 4 viruses were calculated. The results showed that the ENC values of dengue 4 viruses are higher than that of the other subtypes; meanwhile, the GC_3s_ and the ENC values of dengue 4 viruses are all closer to the mean of the human ENC values (Figure 1A). The GC_3s_ values are less than AU_3s_ values for all concerned dengue 4 viruses, and their GC_12_ distributed in a very narrow range (Figure 1B), revealing that the mutations dominated the evolution direction at the third positions of the codons. Based on the theory of mutation pressure, the same occurrence rates of nucleotide mutations were shaped by the same pressure, therefore, a mutational pressure formed by an abundant nucleotide in hosts may lead to a higher occurrence rate of the corresponding nucleotide (Goswami, 2017). The difference between GC_3s_ and AU_3s_ in Figure 1B indicates that the mutation pressure was an obvious factor that influences the codon usage of the dengue 4 viruses. Furthermore, another important codon usage value, the CBI value for each dengue 4 virus, was calculated and compared with the corresponding equalized ENC values (Figure 1C), revealing that there is an obvious negative relationship between them, which could further suggest that the viruses with lower CBI values have higher ENC values.

To further analyze the correlation among all codon usage parameters, correlation coefficients of these numerical values combined with the ENC, GC_12_, GC_3_, etc., were calculated (Supplementary File 3). It had been reported that G/C ending codons are mostly positively correlated with GC_3_ and, accordingly, A/U ending codons are mostly negatively correlated with GC_3_ in most genes, which could be supported by the results obtained in the present study. Significant positive correlations exist between ENC and GC (0.411) and between GC3 content and ENC (0.45), all revealing that the expression level of dengue 4 virus genomes may also be influenced by the patterns of codon usage.

**Figure 2:**
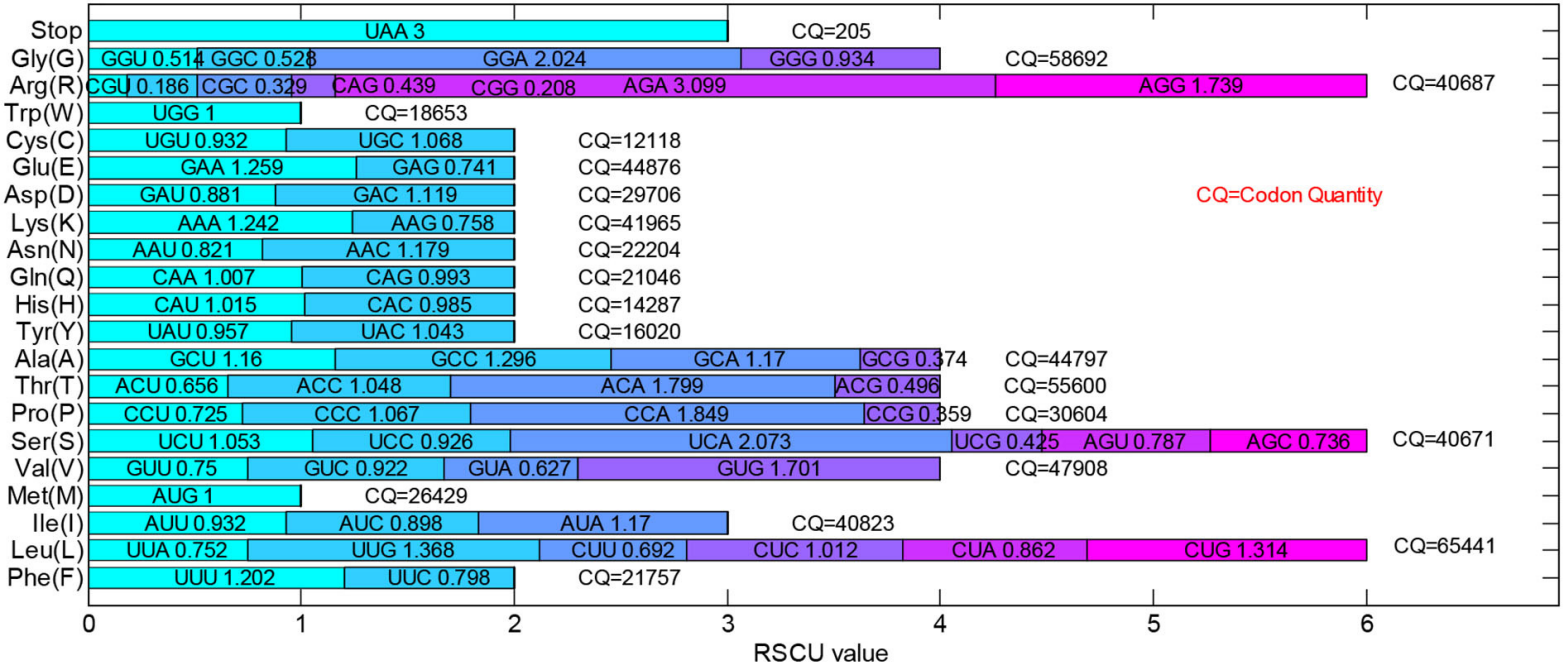
Total relative synonymous codon usage (RSCU) values of the 205 dengue 4 virus genomes. All 205 dengue 4 virus genomes select UAA as the stop codon as the RSCU value of UAA is 3.

The RSCU value, as an important parameter, represents the ratio occurrence frequency of one codon and the expected usage frequency. It is usually used for evaluating the bias of the synonymous codon. The total RSCU values of 205 dengue 4 virus genomes were calculated and shown in Figure 2. If the RSCU value of a codon is >1.0, it would be regarded as a positive codon usage bias. On the contrary, the codons with less RSCU value (<1.0) would be regarded as the less-abundant ones. Especially, the codons, UCG, CCG, ACG, GCG, CGU, CGC, CAG, CGG, GGU, and GGC, whose RSCU values are <0.6, are under-represented codons. The GUG, UCA, CCA, ACA, AGA, AGG, and GGA with high RSCU values (more than 1.6) could be regarded as the over-represented codons. The total RSCU values of dengue 4 virus genomes reflect the overall characteristics of their relative synonymous codon usage pattern. Among 3 stop codons, all sequences select UAA as terminal codon, therefore, there is no RSCU value for UAG and UGA.

### Differences of componential usage pattern between dengue 4 viruses and humans

**Figure 3:**
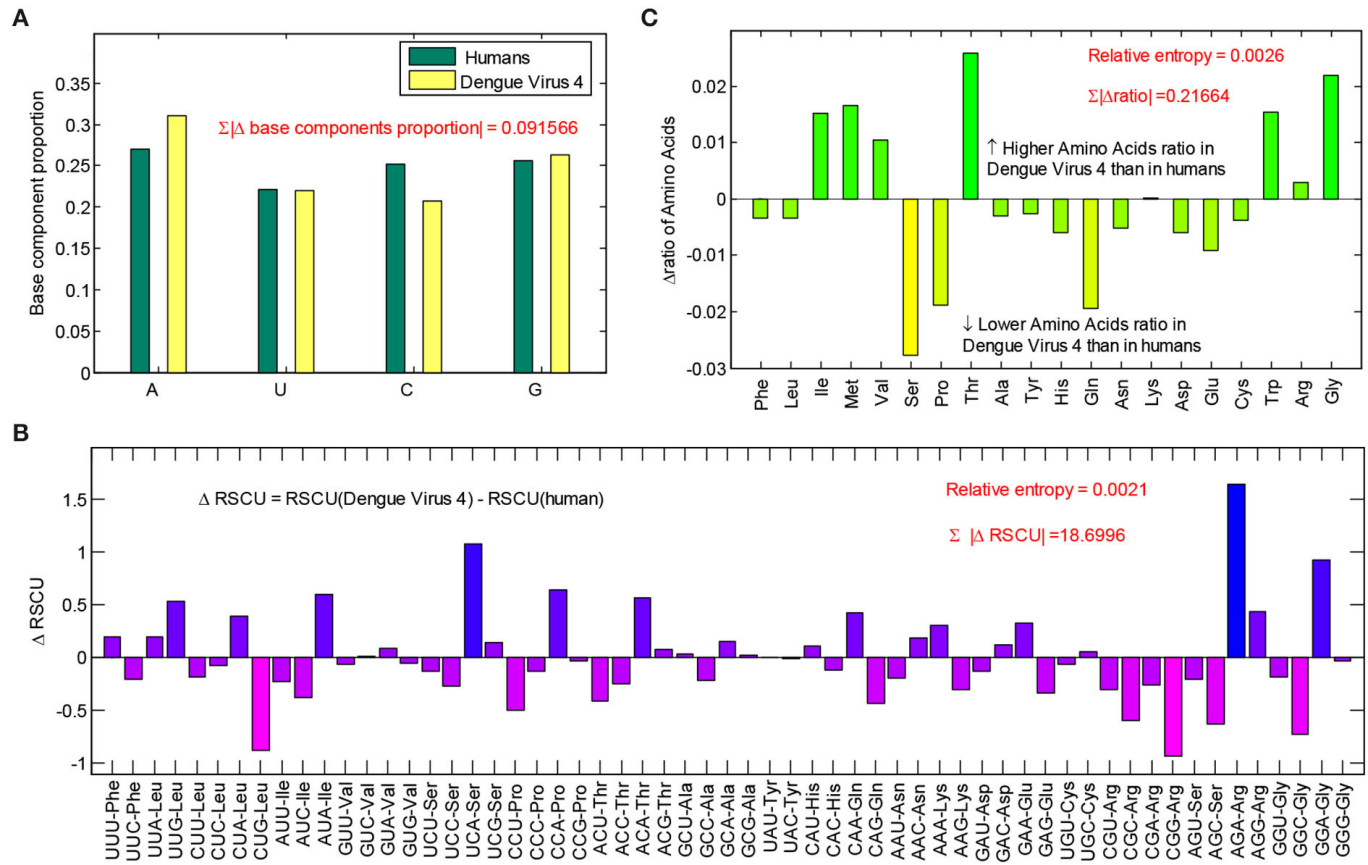
Differences of componential usage pattern between dengue 4 viruses and humans. **(A)** Base usage differences between dengue 4 viruses and humans; **(B)** relative synonymous codon usage (RSCU) differences between dengue 4 viruses and humans; and **(C)** amino acid usage differences between dengue 4 viruses and humans.

The base usage, codon usage, and amino acid usage are the results of a balance between biases generated by mutation and natural selection. To evaluate the effects of human hosts on the componential usage patterns in dengue 4 viruses, differences in componential usage pattern between dengue 4 viruses and humans were further calculated. The differences in base usages showed that A content in dengue 4 viruses is higher than that of humans, C content in dengue 4 viruses is lower than that of humans reversely. Therefore, the A-pressure is the main pressure in dengue 4 viruses. There is a total of 9.16% difference between humans and dengue 4 viruses (Figure 3A) from the base usage level. Differences in relative synonymous codon usages showed that some codons (i.e., UCA, AGA, GGA, CCA, AUA, and ACA) in dengue 4 viruses are much more than those in humans, some codons (i.e., CUG, CCG, GGC, AGC, and CGC) in dengue 4 viruses are much less than those in humans (Figure 3B), which revealed that the A ended codons tend to be optimal ones in dengue 4 viruses. Synonymous mutations in codons could not result in differences in coding proteins; therefore, differences in amino acid usages between dengue 4 viruses and humans were further calculated (Figure 3C). The result showed that about 21.67% of them were different from each other, and Thr, Trp, Gly, Ile, Met, and Val are more optimal amino acids in dengue 4 viruses.

### Evolutionary adaptation to humans of dengue 4 viruses

To further compare the differences between different subtypes of dengue virus and humans, the adaptation of four subtypes of dengue viruses to humans was calculated (Table 1). From the amino acid usage level, the dengue virus 1 is more similar to the humans. However, among four sub-types of dengue viruses, dengue 4 viruses are most similar to human hosts from other levels (i.e., base usage level and relative synonymous codon usage level). The relative entropy between dengue viruses and humans (from dengue viruses to humans) also showed that the dengue 4 viruses are more similar to humans than other subtypes. The evolutionary trends of dengue 4 viruses toward humans are more obvious than other subtypes of dengue viruses on the whole.

**Table 1:** Adaptation of four types of dengue viruses to humans based on the similarity of base usage, relative synonymous codon usage (RSCU), amino acid usage, Kullback–Leibler (K–L) divergences (from dengue viruses to humans) of both RSCU and amino acid ratio.

**Subtypes of dengue viruses**	**Dengue virus 1**	**Dengue virus 2**	**Dengue virus 3**	**Dengue virus 4**
Σ|ΔBase ratio|	0.1027	0.1223	0.1080	0.0916
Σ|ΔRSCU|	20.9550	21.2506	21.0474	18.6996
Σ|Δamino acid ratio|	0.2025	0.2177	0.2259	0.2166
Kullback–Leibler divergence (RSCU)	0.0026	0.0026	0.0026	0.0021
Kullback–Leibler divergence (amino acid ratio)	0.0031	0.0032	0.0031	0.0026

**Figure 4:**
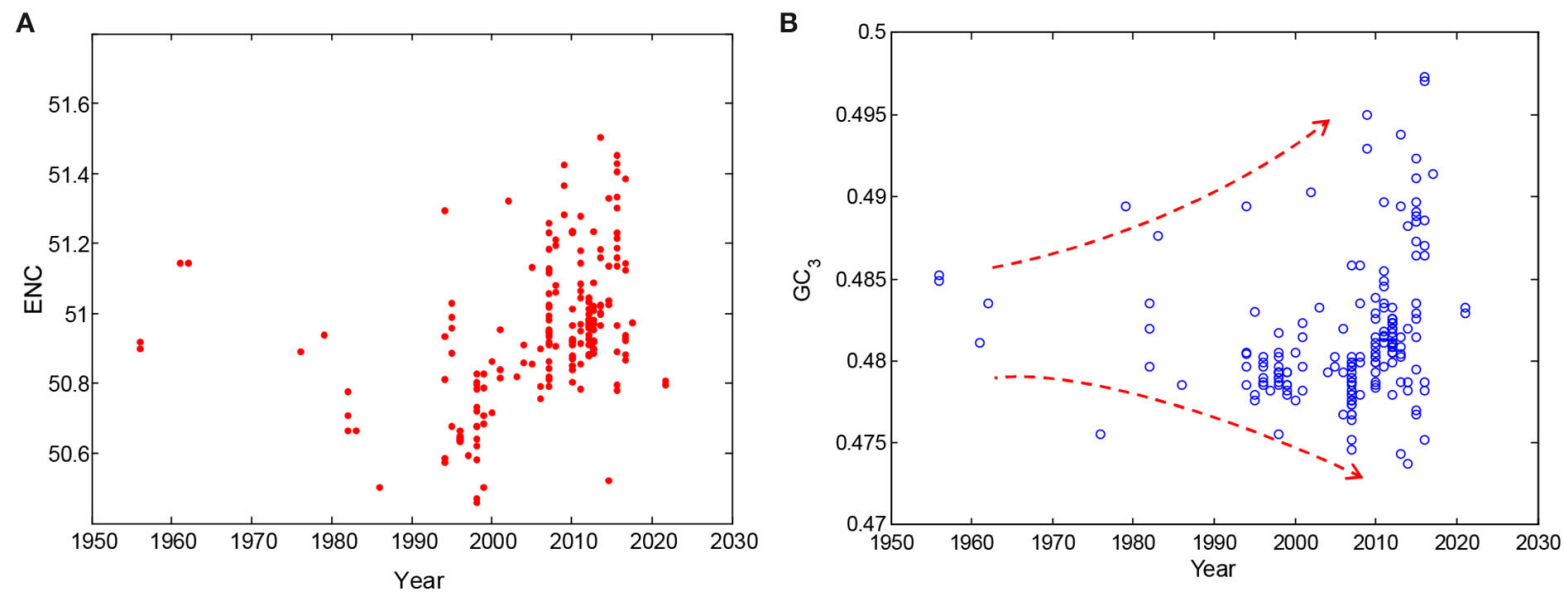
Time-series changes of codon usage pattern in dengue 4 viruses. **(A)** Changes of ENC values in dengue 4 viruses over time; and **(B)** changes of GC_3_ content of dengue 4 viruses over time.

In addition, time-series changes in codon usage pattern in dengue 4 viruses were summarized to illustrate the evolutionary characteristics within them. Although there were fewer samples in the early period (i.e., from 1956 to 1990), the change trend of ENC values showed a significant increase over time, revealing that the mutation pressure was a very important evolutionary pressure (Figure 4A). Changes of GC_12_ were not obvious (Figure 1B), however, the changes of GC_3_ content in dengue 4 virus genomes over time is more broader in their distribution range (Figure 4B), which also showed that mutation plays an important role in their evolution process. The ENC and GC_3s_ values could indicate the further evolutionary direction of the dengue 4 viruses.

## Discussion

Comparative studies on the compositional usage pattern between dengue viruses and humans can explain not only the evolutionary trend of them, but also their basic biological phenomenon at the molecular level. There have been successful attempts to generate attenuated viruses through codon deoptimization (Nogales et al., 2014). The nucleotide biases, especially the unusual codon usage in viruses, could suggest a certain biological function for a viral sequence, and link to mutational events (Li et al., 2018). The ENC value, as an important parameter, plays an important role in interpreting the codon usage pattern. In this work, ENC vs. GC_3s_ of all subtypes of dengue viruses and humans were calculated as shown in Figure 1A to investigate the evolutionary trend in dengue viruses. A larger extent of codon bias in a gene prompts a smaller ENC value (Sueoka, 1999). The details of the distribution of ENC-GC_3s_ values showed that the dengue 4 viruses are of relative lower codon usage bias than other subtypes of dengue viruses, and that the dengue 4 viruses were affected by human-hosts obviously. The neutrality plot (GC_12_ vs. GC_3_) is used to analyze the evolution direction (Tao and Yao, 2020), and to compare the influence extent of natural selection and mutation pressure on codon usage patterns (Tort et al., 2020) of a certain gene. The distribution of GC_12_ rates ranged 0.462–0.465 and the distribution of GC3s ranged 0.475–0.497 (Figure 1B) showed that the GC3s is much more uneven than the GC12s in dengue 4 virus genomes. Previously, researchers have pointed out that the smaller ENC value may denote higher CBI value (Ge et al., 2020). To explore the relationship between the CBI and the ENC, the ENC values were equalized to the distribution range of the CBI (Figure 1C). In the figure, *y* = −0.77x + 0.48 is the linear fitting equation for equalization of ENC vs. CBI, the coefficient −0.77 denotes the strong negative correlation between them, therefore, the fitting result is consistent with the previous theory.

The evolutionary direction of a virus is important for exploring its fitness against human hosts. The RSCU values of a gene represent the relative occurrence frequency of codons (Roy et al., 2021), which were used for evaluating the bias of the synonymous codon. Different virus species may be under different evolutionary pressures. In the present study, mutational A-pressure played an important role in dengue 4 virus genome (Figure 3B), however, the mutational *U*-pressure plays more obvious effect on the first open reading frame (ORF) of coronaviruses (Khrustalev et al., 2020).

The differences between the RSCU values of dengue viruses and the humans could be calculated to reflect its evolutionary characteristics. In the present study, the different values of RSCU between human and dengue 4 viruses suggested that the dengue 4 viruses showed better adaptability to human hosts than other subtypes (Table 1). The codon usage pattern of a virus may evolve toward its host, and in this stage, one base mutation in the coding sequences may not create an effective change in the amino acid sequence. Therefore, non-synonymous changes in dengue viruses were further counted (Figure 3C) to explore its adaptive capacity to the humans. The results showed that the amino acid ratio in the dengue 4 viruses is more similar to that of the human hosts (Table 1), revealing the better adaptiveness in dengue 4 viruses.

The codon usage pattern of viruses may resemble to the host during their evolution process (Kubatko et al., 2016; Diego et al., 2021). To study the codon usage difference between dengue 4 viruses and humans, differences between them were further studied over time to evaluate the potential evolutionary direction (Figures 1A, 4). The codon usage patterns of dengue 4 viruses that emerged recently were more similar to that of humans than those emerged before. Some computational workflows established previously can be generalized and automated to make it applicable in functional prediction (Berkhout and van Hemert, 2015; Abduljalil and Fahd, 2022).

The shorter the coding sequence, the more uneven its compositions because bases or codons may not appear within a short gene sequence. In the present study, all concerned sequences were longer than 10,000 bases; therefore, the componential usage results were of more statistically significant for exploring their overall evolutionary trend. On the contrary, the evolutionary direction of individual genes cannot be explored because of the sequences concerned in the present study were the compounded coding areas for proteins cleaved from the polyprotein. A better understanding of each coding area is also crucial for the development of rapid testing methods of viral serotype (Liberal et al., 2022). Meanwhile, better understanding of the seroprevalence of antibodies against dengue could be used for testing the dengue virus infections (Echegaray et al., 2021). Although the evolutionary trend of the dengue virus 4 was highlighted in the present study, the coding areas for structural proteins and non-structural proteins still need further research respectively.

Characteristics of the spatiotemporal patterns of dengue epidemic, for instance, the 2019 dengue epidemic in Bhutan (Tsheten et al., 2021) and dengue epidemic from 2007 to 2019 in Sri Lanka (Prabodanie et al., 2020), were closely concerned (Anwar et al., 2022). In the current study, however, all data on the dengue virus 4 were considered to explicit their long-term evolutionary features. With the emergence of diverse dengue viruses in the future, epidemiological characteristics and the evolutionary trend of their genomes needs to be further studied in the future (Yue et al., 2021), meanwhile, further research should emphasize on the genetic diversity in these emerging viruses.

## Conclusion

Dengue fever is a severe illness that affects infants, young children, and adults, and it has spread around the world. Many risk assessments and guidelines have been put forward by many countries to prevent the spread of dengue virus. As an important genotype of the dengue virus, it occurred in many places. In this study, base usage, codon usage, and amino acid usage characteristics were used to explore the genetics of dengue 4 viruses. Not only the ENC-plot, RSCU values of dengue 4 viruses were studied, but also the characteristics of their distances to humans were analyzed. The evolution trend of dengue 4 viruses was independent of the passage of time. In conclusion, dengue 4 viruses adapt to humans better than the other subtypes. Our findings could also provide a comprehensive assessment of the adaptation of other human-host viruses.

## Data availability statement

The original contributions presented in the study are included in the article/Supplementary material, further inquiries can be directed to the corresponding authors.

## Author contributions

GL and LS conceived and designed the work and drafted the manuscript. GL and LZ conducted all bioinformatics analyses and arranged figures and tables. GL, LS, and BX revised the manuscript, read, and approved the final manuscript. All authors contributed to the article and agreed to the submitted version.

## Funding

This research work was funded by the Special Scientific Research Project of Education Department of Shaanxi Province, grant number 18JK0377.

## Conflict of interest

The authors declare that the research was conducted in the absence of any commercial or financial relationships that could be construed as a potential conflict of interest.

## Publisher's note

All claims expressed in this article are solely those of the authors and do not necessarily represent those of their affiliated organizations, or those of the publisher, the editors and the reviewers. Any product that may be evaluated in this article, or claim that may be made by its manufacturer, is not guaranteed or endorsed by the publisher.

## Supplementary material

Supplementary File 1 Dengue viruses information.

Supplementary File 2 The relative synonymous codon usage (RSCU) values of humans.

Supplementary File 3 Correlations among codon usage patterns.
