# Supplementary material for: Componential usage patterns in dengue 4 viruses reveal their better evolutionary adaptation to humans
Source: Front Microbiol. 2022 Sep 20;13:935678. doi: 10.3389/fmicb.2022.935678 (PMC9530264; doi:10.3389/fmicb.2022.935678)
Supplement: Supplementary File 3 — Correlations among codon usage patterns. [file Data_Sheet_3.doc]

Supplementary File 3: Correlations among codon usage patterns

**Componential Usage Patterns in Dengue 4 Viruses Reveal Their Better Evolutionary Adaptation to Humans**

Gun Li ^a,*^, Liang Shi ^a, b,*^, Liang Zhang ^a^, Bingyi Xu ^a^

a Laboratory for Biodiversity Science, Department of Biomedical Engineering, School of Electronic Information Engineering, Xi'An Technological University, Xi'An, Shaanxi, China

b  Key Laboratory of Analytical Chemistry for Life Science of Shaanxi Province, School of Chemistry and Chemical Engineering, Shaanxi Normal University, Xi’An, Shaanxi, China

**Correspondence:**

Gun Li (ligun@xatu.edu.cn)

Liang Shi (shiliang@xatu.edu.cn)

Figure. CorrelationS of codon usage patterns in dengue 4 virus genomes
